# Bias Mitigation in Primary Health Care Artificial Intelligence Models: Scoping Review

**DOI:** 10.2196/60269

**Published:** 2025-01-07

**Authors:** Maxime Sasseville, Steven Ouellet, Caroline Rhéaume, Malek Sahlia, Vincent Couture, Philippe Després, Jean-Sébastien Paquette, David Darmon, Frédéric Bergeron, Marie-Pierre Gagnon

**Affiliations:** 1 Faculté des sciences infirmières, Université Laval Québec, QC Canada; 2 Vitam Research Center on Sustainable Health Québec, QC Canada; 3 Département de médecine familiale et de médecine d'urgence de la Faculté de médecine, Université Laval Québec, QC Canada; 4 Research Center of Quebec Heart and Lungs Institute Québec, QC Canada; 5 École Nationale des Sciences de l’Informatique, Université de La Manouba La Manouba Tunisia; 6 Département de physique, de génie physique et d'optique de la Faculté des sciences et de génie, Université Laval Québec, QC Canada; 7 Risques, Epidémiologie, Territoires, Informations, Education et Santé. Département d’enseignement et de recherche en médecine générale, Université Côte d'Azur Nice France; 8 Direction des services-conseils de la Bibliothèque, Université Laval Québec, QC Canada

**Keywords:** artificial intelligence, AI, algorithms, expert system, decision support, bias, community health services, primary health care, health disparities, social equity, scoping review

## Abstract

**Background:**

Artificial intelligence (AI) predictive models in primary health care have the potential to enhance population health by rapidly and accurately identifying individuals who should receive care and health services. However, these models also carry the risk of perpetuating or amplifying existing biases toward diverse groups. We identified a gap in the current understanding of strategies used to assess and mitigate bias in primary health care algorithms related to individuals’ personal or protected attributes.

**Objective:**

This study aimed to describe the attempts, strategies, and methods used to mitigate bias in AI models within primary health care, to identify the diverse groups or protected attributes considered, and to evaluate the results of these approaches on both bias reduction and AI model performance.

**Methods:**

We conducted a scoping review following Joanna Briggs Institute (JBI) guidelines, searching Medline (Ovid), CINAHL (EBSCO), PsycINFO (Ovid), and Web of Science databases for studies published between January 1, 2017, and November 15, 2022. Pairs of reviewers independently screened titles and abstracts, applied selection criteria, and performed full-text screening. Discrepancies regarding study inclusion were resolved by consensus. Following reporting standards for AI in health care, we extracted data on study objectives, model features, targeted diverse groups, mitigation strategies used, and results. Using the mixed methods appraisal tool, we appraised the quality of the studies.

**Results:**

After removing 585 duplicates, we screened 1018 titles and abstracts. From the remaining 189 full-text articles, we included 17 studies. The most frequently investigated protected attributes were race (or ethnicity), examined in 12 of the 17 studies, and sex (often identified as gender), typically classified as “male versus female” in 10 of the studies. We categorized bias mitigation approaches into four clusters: (1) modifying existing AI models or datasets, (2) sourcing data from electronic health records, (3) developing tools with a “human-in-the-loop” approach, and (4) identifying ethical principles for informed decision-making. Algorithmic preprocessing methods, such as relabeling and reweighing data, along with natural language processing techniques that extract data from unstructured notes, showed the greatest potential for bias mitigation. Other methods aimed at enhancing model fairness included group recalibration and the application of the equalized odds metric. However, these approaches sometimes exacerbated prediction errors across groups or led to overall model miscalibrations.

**Conclusions:**

The results suggest that biases toward diverse groups are more easily mitigated when data are open-sourced, multiple stakeholders are engaged, and during the algorithm’s preprocessing stage. Further empirical studies that include a broader range of groups, such as Indigenous peoples in Canada, are needed to validate and expand upon these findings.

**Trial Registration:**

OSF Registry osf.io/9ngz5/; https://osf.io/9ngz5/

**International Registered Report Identifier (IRRID):**

RR2-10.2196/46684

## Introduction

Developments in computer science have led to artificial intelligence (AI) models that learn from large datasets and can perform independent analysis [1-4]. Significant progress has been made in these tasks with the development of machine learning (ML). This branch of AI focuses on understanding, generating, and reasoning based on data without explicit human instructions [2,3] Such ML algorithms use datasets known as “training datasets” to capture the patterns required for clustering tasks or predictive modeling [3,4]. These models are now used in multiple contexts and industries to predict the likelihood of an event or to support human decision-making [4]. In health care, AI models applied in radiology can potentially detect and predict the progression of cancerous tumors accurately [5]. Algorithms can also be useful in community-based primary health care (CBPHC) for identifying individuals, such as heart failure or diabetes outpatients, who require specific health care services [6]. As defined by the Canadian Institutes of Health Research, CBPHC encompasses a comprehensive array of services aimed at community well-being, including primary prevention (such as public health), health promotion, disease prevention, diagnosis, treatment, and management of chronic and episodic illnesses, rehabilitation support, and end-of-life care [7].

Despite the potential benefits of AI, such as compensating for workforce shortage and maximizing access to CBPHC [6], algorithm biases toward diverse groups can hinder their application in health care settings. These biases may be perpetuated when protected attributes [1], as identified by the place of residence, race/ethnicity/culture/language, occupation, gender/sex, religion, education, socioeconomic status, and social capital (PROGRESS-Plus) framework [8], are underrepresented or misrepresented in the training data of algorithms [1,9]. Strategies aimed at identifying and mitigating bias, defined as a persistent inclination either in favor or toward something [9], in predictive models are in development and beginning to be empirically applied [10,11]. In computer science, attempts to achieve algorithmic fairness can involve which are (1) preprocessing, (2) in-processing, or even, (3) postprocessing strategies, such as those used in “out-of-the-box” commercial AI models [4]. Academic disciplines beyond computer science, such as medicine, management, and ethics, are also closely involved in addressing issues related to identifying potential bias toward diverse groups in AI models [1,3]. However, there remains a knowledge gap regarding which strategies and methods have been empirically applied to mitigate bias toward diverse groups in CBPHC algorithms [10,12].

To address this gap, we conducted a scoping review aimed at identifying and describing (1) the attempts made to mitigate bias in primary health care AI models, (2) which diverse groups or protected attributes have been considered, and (3) the results regarding bias attenuation and the overall performance of the models.

## Methods

### Search Strategy

We conducted a scoping review informed by the Joanna Briggs Institute (JBI) [13] and used the Population (or Participant), Concept, and Context Framework [14], as shown in Table 1.

**Table 1:** Population (or Participant), Concept, and Context framework used for the search strategy.

PCC^a^ elements [14]	Definition (per JBI^b^ Reviewer’s Manual)	PCC elements applied in this review
Population	“Important characteristics of participants, including age and other qualifying criteria” (11.2.4)	Any diverse groups [8] based on their personal or protected attributes [1].
Concept	“The core concept examined by the scoping review should be clearly articulated to guide the scope and breadth of the inquiry. This may include details that pertain to elements that would be detailed in a standard systematic review, such as the “interventions” or “phenomena of interest” (11.2.4)	Strategies, attempts, or methods for assessing and mitigating bias in artificial intelligence.
Context	“May include...cultural factors such as geographic location or specific racial or gender-based interests. In some cases, context may also encompass details about the specific setting.”	Community-based primary health care [7].

^a^PCC (Population [or Participant], Concept, and Context) framework [14].

^b^JBI: Joanna Briggs Institute.

### Bias Mitigation in Primary Health Care Artificial Intelligence Models

Primary review questions are (1) What attempts have been made to mitigate bias in primary health care AI models? (2) Which diverse groups or protected attributes have been considered? and (3) What are the results regarding bias attenuation and model performance?

In November 2022, we developed a search strategy aligned with the main concepts of our primary review questions with an experienced librarian in 4 relevant databases (MEDLINE [Ovid], CINAHL [EBSCO], PsycInfo [Ovid], and Web of Science). The results of the search strategy in Web of Science were limited to the following 2 indexes: Science Citation Index Expanded and Emerging Sources Citation Index. We used 5 relevant articles to test the sensitivity of our search strategy, focusing on peer-reviewed publications from the past 5 years (between January 1, 2017, and November 15, 2022). The search strategies for each database can be found in Multimedia Appendix 1.

### Data Collection

We imported all sources (n=1603) into the web-based collaborative tool Covidence (Veritas Health Innovation) [15], which automatically identified and removed 581 duplicates, with an additional 4 removed manually. The inclusion and exclusion criteria are presented in Table 2. During the title and abstract screening phase, 7 reviewers independently assessed the abstracts based on the selection criteria. We piloted the screening process on 50 sources that all reviewers independently assessed. Reviewers included a source if it met our inclusion criteria, such as featuring an AI predictive model in health, targeting primary health care populations, and presenting a strategy or method for reducing bias. All titles and abstracts were screened independently by at least 2 reviewers, with any discrepancies resolved through consensus involving all reviewers, including at least 1 senior researcher.

**Table 2:** Inclusion and exclusion criteria.

PCC (Population, Concept, and Context) elements [14]	Inclusion criteria	Exclusion criteria
Population	Any populations targeted by CBPHC^a^ interventions.	Any populations targeted by hospital or specialized care interventions.
Concept	All methods or strategies deployed to assess and mitigate bias toward diverse groups or protected attributes in AI models. All mitigation methods or strategies deployed to promote and increase equity, diversity, and inclusion in CBPHC algorithms.	Methods or strategies deployed to assess and mitigate bias in the AI model itself (eg, biased prediction of treatment effects), rather than bias related to individuals’ characteristics or protected attributes. Strategies, methods, or interventions that are not related to CBPHC. CBPHC interventions that do not include any algorithm or AI system.
Context	Include all CBPHC algorithms (AI) applications that can perpetuate or introduce potential biases toward diverse groups based on their characteristics or protected attributes.	Algorithms used by primary health care providers for support in administrative tasks and operational aspects, rather than for clinical decisions.
Study design, study type, and time frame	All empirical studies published in English or French between 2017 and 2022.	Reviews, opinions, commentaries, editorial content, conference papers, communications, protocols, magazine articles, and so on.

^a^CBPHC: Community-based primary health care.

For the remaining articles assessed for eligibility at the full-text review stage, we searched for and obtained any missing full texts of selected references, then imported them into Covidence. Out of 5 reviewers independently applied the same selection criteria, and all reasons for exclusion were recorded in Covidence. All full texts underwent dual screening. As in the previous stage, any discrepancies regarding the included studies were resolved through consensus among all reviewers, including at least one senior researcher.

### Data Extraction

One experienced reviewer performed the extraction of the included studies, and 2 senior researchers validated the data for all of them. We also hand-searched [16] and identified 2 relevant articles [17,18] related to 2 included studies [19,20], which were added to Covidence for extraction. Based on reporting standards for AI in health care [21], we extracted the following information (title of the paper, year of publication, lead author, and country), study objective, discipline and study design, AI model features, study population and setting, AI model architecture and evaluation, bias assessment method, strategy for deployment, diverse groups concerned, bias mitigation results, and the impact on AI model performance and accuracy.

### Quality Assessment

One senior reviewer appraised the quality of the included studies by applying the Mixed-Methods Appraisal Tool (MMAT) [22,23] and at least one senior researcher validated each of them.

### Data Synthesis

In accordance with the JBI recommendations [24], we synthesized data using structured narrative summaries around our review concepts (eg, model data source, model input, model output, diverse groups, or protected attributes), mitigation strategies deployed, and the results on bias mitigation and overall model performance. We reported our findings based on the PRISMA-ScR (Preferred Reporting Items for Systematic Reviews and Meta-Analyses extension for Scoping Reviews) [25].

### Ethical Considerations

We obtained approval from the ethics board of the “Comité d’éthique de la recherche sectoriel en Santé des Populations et Première Ligne du Centre Intégré Universitaire de Santé et de Services Sociaux de la Capitale-Nationale” for the Protecting and Engaging Vulnerable Populations in the Development of Predictive Models in Primary Health Care for Inclusive, Diverse and Equitable AI (PREMIA) project (#2023-2726).

## Results

**Figure 1:**
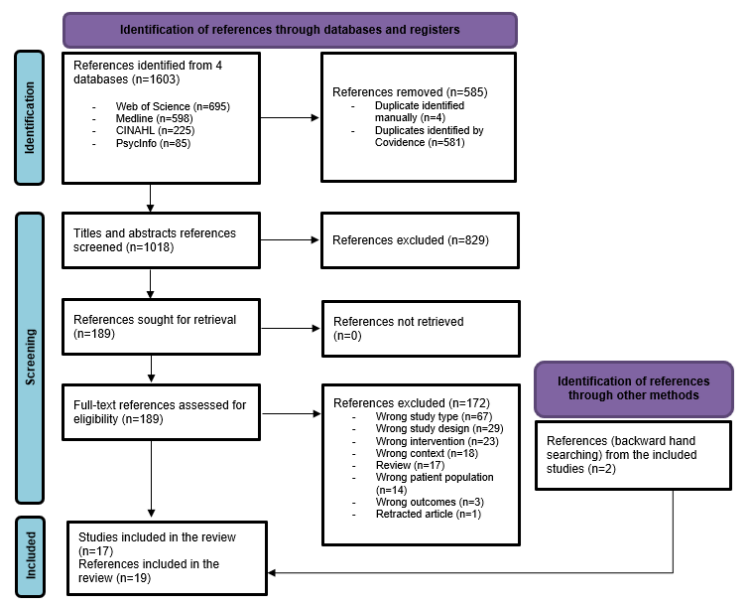
PRISMA (Preferred Reporting Items for Systematic reviews and Meta-Analyses) flow diagram.

Out of a total of 1018 titles and abstracts, along with 189 full-text articles that underwent dual screening, 17 studies [19,20,26-40] met our eligibility criteria. The PRISMA (Preferred Reporting Items for Systematic Reviews and Meta-Analyses) 2020 flow diagram is shown in Figure 1 [41].

The relatively high number of exclusions at the full-text review stage (172/189, 91%) can be attributed to our inclusive approach in the previous stage. For example, some reviews (17/189, 9%) and incorrect study types (67/189, 35%), such as editorials, commentaries, or conference papers, were excluded at this stage. Other exclusion reasons (88/189, 47%) included models that lacked AI components, models focusing on health care operational processes (eg, workflow modeling), studies targeting populations receiving specialized care (eg, hospitalized or cancer patients), interventions such as imaging research that were outside the scope of CBPHC, and methods for mitigating bias that were applied to the AI model itself (eg, biased predictions of treatment effects) rather than addressing biases related to diverse groups or personal attributes.

### Overview of Included Studies

Of the 17 included studies published between 2019 and 2022, we identified 7 studies in the discipline of data science or informatics, 7 in medical informatics, 1 in medical ethics and informatics, 1 in medical ethics using a Delphi method, and 1 in management care ethics using a user-centered design. Most studies have been conducted in the United States (15/17, 88%), 1 in the United Kingdom, and 1 in Italy. The main characteristics of the included studies can be found in Multimedia Appendix 2.

### Quality Assessment of Included Studies

Most studies had a quantitative descriptive study design (14/17, 82%), while 2 used a mixed methods design, and 1 used a qualitative design. All studies showed high quality, receiving scores of 3 or 4 stars (on a possibility of 5). All MMAT scores can be found in Multimedia Appendix 3.

### Diverse Groups Considered

The most frequently studied protected attributes were race (or ethnicity), examined in 71% (12/17) of studies, and sex (defined as binary male versus female), considered in 59% (10/17) of studies. None of the studies distinguished between biological sex and socially constructed gender, and 5 of them incorrectly identified sex as gender. Race or ethnicity was most often categorized as White or Black, Black or non-Black or, in one study, as Asian, Black, White, and other.

Other protected attributes considered by the studies included age (7/17, 41%), socioeconomic status or its proxies, such as income, work class, education, health care insurance (5/17, 29%), place of residence (2/17, 12%), marital status (1/17, 6%), and disability status (1/17, 6%).

### Categorization of Deployed Bias Mitigation Strategies

We identified considerable heterogeneity across the studies, which used various strategies and methods to assess and mitigate bias in algorithms impacting diverse groups. We categorized these efforts into four groups: (1) addressing bias in existing AI models or datasets, (2) mitigating biases from data sources such as electronic health records (EHRs), (3) developing tools that incorporate a “human-in-the-loop” approach, and (4) identifying ethical principles to guide informed decision-making.

### Attempts in Existing AI Models or Datasets

We identified 7 studies that attempted to mitigate biases in existing AI models or datasets [19,20,27,28,35,37,39].

A debiasing attempt was made on an insurance coverage algorithm designed to identify individuals who could benefit from health resources according to their health needs [35]. Risk scores were initially calculated based on projected future costs rather than uncontrolled or unmanaged illnesses, disadvantaging Black patients. By changing the data labeling to focus on future illness rather than future costs, the percentage of Black patients who could benefit from health resources increased significantly [35].

Another cohort study [37] using a Medicaid enrollees’ dataset showed that reweighing was more effective at reducing bias in postpartum depression risk scores between White and Black individuals compared with training without the race variable for comparison. Initially, it was found that the White individuals had higher rates of postpartum depression and mental health service use. However, after comparing postpartum depression rates between races based on population surveys, it became clear that the higher rates in White women might be due to disparities in the timely assessment, screening, and detection of symptoms in Black women [37].

A total of three other studies include (1) retraining models with data that incorporated health equity measures resulted in a slight decrease in performance for detecting abnormal electrocardiograms but significantly reduced gender, race and age biases [19]; (2) increasing diversity in the training data of a predictive pulmonary disease model improved its performance [27]; and (3) although a mental health assessment model achieved high accuracy, its performance was statistically higher and more accurate for men than for women [18]. The use of an algorithmic disparate remover, by adjusting the modeling data, significantly reduced this disparity while maintaining high accuracy [20].

Another attempt to assess bias involved replicating models predicting liver disease [39]. Importing an existing dataset reproduced predictive models with high accuracy but revealed a previously unobserved bias, with women experiencing a higher false negative rate.

We identified only 1 in-processing debiasing attempt [28]. Out of 2 algorithmic fairness strategies, group recalibration and equalized odds, were used to recalibrate a predictive model of cardiovascular diseases that was not initially adjusted for attributes such as sex or race. This resulted in an exacerbation of false positive and negative rates differences between groups, as well as overall model miscalibration.

### Attempts in Data Sourcing

We identified 5 studies that attempted to mitigate biases in data sourcing [26,31,32,38,40].

Based on published synthetic datasets, such as the analysis of the American Time Use Survey dataset, using fairness metrics revealed potential discrepancies in representativeness between real and synthetic data across age, sex, and race [26].

Out of 4 other studies investigated EHRs datasets [31,32,38,40]. A natural language processing model was developed to extract vital sign features from unstructured notes, comparing risk scores with 2 convenience samples. This method reduced the missingness of vital signs by 31%, thereby mitigating possible discrimination toward diverse groups, such as Black men or Black women [32]. Based on data from a previous study, 2 ML models were trained to compare balanced error rates across different socioeconomic status levels and the incompleteness of EHRs data [31]. Asthmatic children with lower socioeconomic status exhibited larger balanced error rates than those with higher socioeconomic status and had more missing information regarding asthma care, severity, or undiagnosed asthma, despite meeting asthma criteria [31].

Potential bias based on place of residence in EHRs was examined by 2 studies [38,40]. Rebalancing class labels by adding zip-code level information to 19,367 EHRs during the preprocessing step showed no significant deviation in performance, indicating that bias can be mitigated through preprocessing [38]. Meanwhile, a simple 30-day readmission prediction model was developed, categorizing each patient as local (nearby) or not (far) [40]. The performance with and without this variable was assessed, revealing no significant differences. Considering that living locally only affects the observability of the outcome (eg, a patient may be readmitted to a different hospital), differential bias assessment cannot rely solely on observed data [40].

### Attempts in Developing Tools With a “Human-in-the-Loop” Approach

We identified 3 studies that attempted to mitigate biases by incorporating a “human-in-the-loop” approach [29,30,36].

These studies led to the development of “human-in-the-loop” tools: (1) a visual tool for auditing and mitigating bias from tabular datasets, which was tested through experiments on 3 datasets with user participation and significantly reduced bias compared with another commercial debiasing toolkit [29]; (2) pragmatic tools developed for better use of risk scores with a Medicare members’ dataset, allowing users to identify appropriate risk scores for each subgroup to achieve equality of opportunity [30]; and (3) a tool called “FairLens” capable of identifying and explaining biases, which was tested using a fictitious black box model serving as a decision support system [36]. Empirically validated by injecting biases into this fictitious decision support system, this tool outperformed other standard measures and enabled experts to identify problematic groups or affected patients, thereby allowing for the detection of potential misclassification [36].

### Attempts at Identifying Ethical Principles for Informed Decision-Making

We identified 2 empirical studies that attempted to mitigate biases by identifying ethical principles for informed decision-making [33,34].

To assess the potential missingness of EHR data from phenotyping technology, a Delphi study was conducted to address ethical challenges and reach a consensus on the importance of privacy, transparency, consent, accountability, and fairness [33]. In addition, a user-centered design study was conducted to identify user requirements, mainly intended for health managers and clinicians, to support informed decision-making and confidence in using a hepatitis C severity illness predictive model prototype [34].

## Discussion

### Principal Findings

The reviewed studies illustrate a multifaceted approach to mitigating bias in primary care AI models. Strategies include retraining, reweighing, relabeling, adding more diversity, and attempting to replicate existing modeling data [19,20,27,35,37,39], as well as algorithmic recalibration applied to an existing prediction model [28]. Other strategies involve the development and application of fairness metrics to ensure equitable distributions in previously published databases [26], and the identification of missingness in EHRs datasets by rebalancing class labels or adding information [31,32,38]. Another group of strategies includes the introduction of visual interactive tools for human-in-the-loop bias auditing [29,30,36]. All these attempts cover a broad spectrum of interventions, ranging from data preprocessing and algorithmic modification to post hoc analysis, demonstrating the complexity and variety of approaches needed to address bias in AI models in primary health care.

The studies collectively address a wide range of protected attributes [1,8], including race or ethnicity [19,26,28-37], sex [19,20,26-31,36,39], age [19,26,27,29-31,36], socioeconomic status (SES) [27,29,31,33,36], and other demographic variables such as place of residence [38,40]. This underlines the recognition of the multifaceted nature of bias, which can intersect across various dimensions of identity and social determinants of health [9,42]. However, we have identified disparities in the number of protected attributes studied. Race (White vs Black) and sex (male vs female) are most frequently investigated, whereas other attributes, such as disability and gender, are underresearched or not studied at all.

Bias mitigation efforts reveal a nuanced landscape where attempts to reduce bias across protected attributes can result in complex trade-offs with model performance. For example, a decrease in overall model performance accompanied by significant reductions in bias was observed following the implementation of constrained optimization [19]. Similarly, improvements in calibration for specific groups came at the cost of increased disparities in false positive and false negative rates between groups [28]. Despite these trade-offs, the efforts have largely been successful in reducing bias, as evidenced by a study that achieved fairer distributions in synthetic data [26], and in another study where human-in-the-loop interventions significantly reduced bias while maintaining utility [29].

These empirical findings reinforce theoretical insights that emphasize the importance of health equity between protected and unprotected attributes [1,8]. To mitigate bias in AI health models, distributive justice options for ML have been proposed: (1) equal patient outcomes; (2) equal performance; and (3) equal allocation of resources [1]. Since these different types of fairness options are often incompatible, optimizing all these parameters seems challenging, as demonstrated by an identified study [28]. Trade-offs are essential, and a participatory process involving key stakeholders, including ethicists, clinicians, and marginalized populations, is strongly encouraged [1]. While striving to create ethically robust AI models, selected studies often reveal tension, as efforts to reduce bias can sometimes lead to a decrease in the model’s overall performance. This presents a critical challenge: balancing the imperative of fairness with the need to maintain high accuracy and efficiency in algorithmic outputs.

### Comparison With Previous Work

Initiatives focused on the fair use of AI in health care and the assessment of bias risk in AI predictive models have been published in recent years. Notable initiatives include Consolidated Standards of Reporting Trials-Artificial Intelligence (CONSORT-AI) and Standard Protocol Items Recommendations for Interventional Trials-Artificial Intelligence (SPIRIT-AI) [43], which provide guidelines for the ethical presentation of the results of trials conducted with AI in the health care field. To assess the risk of bias in diagnostic and prognostic prediction model studies, the “Prediction Model Risk of Bias Assessment Tool” (PROBAST) [44] can be used. PROBAST consists of a list of signaling questions grouped into 4 categories: participants, predictors, outcomes, and analysis. This tool was used in a systematic scoping review to assess the quality of primary studies reporting applications of AI in CBPHC [45].

However, the objective of our scoping review differs; it is not to identify biases in the AI prediction models themselves, but rather to examine biases toward groups that are underrepresented or misrepresented in these AI models. An identified review has used and adapted PROBAST to assess related protected attributes, but the AI predictive models studied were hospital-based and not relevant to primary care [11]. We also identified a scoping review protocol that focused on bias toward diverse groups in AI systems in primary care; however, unless we are mistaken, the results of this protocol have never been published [10]. Another identified review aimed to assess age-related bias in AI but did not focus on primary health care [46]. Finally, we identified another systematic review investigating health inequities in primary care, but it adopted a system-wide perspective, focusing on aspects such as patient consultation and effects on health systems [47].

To our knowledge, no other published review has the objectives of identifying (1) the bias mitigation strategies or methods in primary health care, (2) the diverse groups that are underrepresented or misrepresented, and (3) the results of bias mitigation and AI model performance.

### Strengths and Limitations

The strengths of this review include results that can be translated into recommendations for various stakeholders, such as AI developers, researchers, and decision makers. However, we acknowledge some limitations. First, we limited our search strategy to the last 5 years before November 2022 and focused on 4 databases, which may have excluded some relevant studies. Second, the extraction of studies and quality assessment were conducted only once, although all of them were validated by at least one senior researcher. Third, due to the heterogeneity of the studies, we were unable to combine results through a quantitative synthesis and remained at a narrative level of reporting. Finally, our review primarily identified research from a North American setting, which reduces its transferability to other continents.

### Future Directions and Dissemination Plan

This scoping review serves as the initial phase of the iterative project “Protecting and Engaging Vulnerable Populations in the Development of Predictive Models in Primary Health Care for Inclusive, Diverse, and Equitable AI” (PREMIA).

Following the results of this review, we have developed a framework currently validated by a diverse group of experts, including clinicians, public health managers, primary care researchers, data scientists, and patient and citizen partners. This group is concentrating on existing AI predictive models and the bias mitigation strategies identified in our scoping review. Diverse populations, such as older adults, individuals with disabilities, and people from various racial and ethnic backgrounds, are actively involved in this second phase of PREMIA. We plan to prepare and submit a manuscript based on the findings of this Delphi study.

In addition, in recognition of the rapid advancements in this field, we plan to update this literature review in 2027 using a similar search strategy. This iterative approach will allow us to refine our framework and track the progress of bias mitigation in AI models within primary health care. Indigenous peoples in Canada represent a group historically underrepresented in health research, leading to inequities [3]. Since no other study has addressed bias related to Indigenous status, we collaborate with Indigenous representatives to develop methods for mitigating this bias in CBPHC algorithms.

### Conclusion

This review identifies strategies and methods for mitigating bias in primary health care algorithms, considers diverse groups based on their personal or protected attributes, and examines the results of bias attenuation and model performance. The findings suggest that biases toward diverse groups can be more effectively mitigated when data are open-sourced, multiple stakeholders are involved, and during the preprocessing stage of algorithm development. More empirical studies are needed, with a focus on including participants who embrace greater diversity, such as nonbinary gender identities or Indigenous peoples in Canada.

## Appendix

Multimedia Appendix 1 Database Search Strategies.

Multimedia Appendix 2 Characteristics of Included Studies.

Multimedia Appendix 3 Quality assessment: MMAT (Mixed-Methods Appraisal Tool) scores.

Multimedia Appendix 4 PRISMA-ScR checklist.

## Abbreviations

AI: artificial intelligence
CBPHC: community-based primary health care
CONSORT-AI: Consolidated Standards of Reporting Trials-Artificial Intelligence
EHR: electronic health record
JBI: Joanna Briggs Institute
ML: machine learning
MMAT: Mixed-Methods Appraisal Tool
PREMIA: Protecting and Engaging Vulnerable Populations in the Development of Predictive Models in Primary Health Care for Inclusive, Diverse and Equitable AI
PRISMA: Preferred Reporting Items for Systematic reviews and Meta-Analyses
PRISMA-ScR: Preferred Reporting Items for Systematic reviews and Meta-Analyses extension for Scoping Reviews
PROBAST: Prediction model Risk Of Bias Assessment Tool
PROGRESS: Place of residence, race/ethnicity/culture/language, occupation, gender/sex, religion, education, socioeconomic status, and social capital
SES: socioeconomic status
SPIRIT-AI: Standard Protocol Items Recommendations for Interventional Trials-Artificial Intelligence

## Data Availability

All data generated or analyzed during this study are included in this published article and Multimedia Appendices 1-3.
