# Supplementary material for: Bias Mitigation in Primary Health Care Artificial Intelligence Models: Scoping Review
Source: J Med Internet Res. 2025 Jan 7;27:e60269. doi: 10.2196/60269 (PMC11751650; doi:10.2196/60269)
Supplement: Multimedia Appendix 1 [file jmir_v27i1e60269_app1.pdf]

Databases search strategy

Medline (OVID)

Date of the search: 15-11-2022
Database limit: The results of the research strategy were limited to the last 5 years (2017-2022)

#	Search strategy	Results
1	Artificial Intelligence/ OR exp "Machine Learning"/ OR exp Neural Networks, Computer/ OR Expert Systems/ OR Natural Language Processing/ OR Algorithms/	375775
2	Expert System?.ti,ab,kw,kf OR ((artificial OR computational) adj2 intelligence).ti,ab,kw,kf OR ((Machine? OR Supervised OR Unsupervised OR Reinforcement OR Deep) adj2 Learning).ti,ab,kw,kf OR "Neural Networks".ti,ab,kw,kf OR (AI adj (technolog* OR system? OR algorith* OR application?)).ti,ab,kw,kf OR (predicti* adj (model? OR algorithm?)).ti,ab,kw,kf OR "natural language processing".ti,ab,kw,kf	213938
3	1 OR 2	504772
4	Bias/	25839
5	Bias.ti,ab,kw,kf OR biases.ti,ab,kw,kf	239393
6	4 OR 5	250499
7	Community Health Services/ OR Primary Health Care/ OR Delivery of Health Care/ OR Healthcare Disparities/ OR Health Equity/ OR Decision Support Systems, Clinical/ OR Decision Support Techniques/	275373
8	"Decision Support".ti,ab,kw,kf OR ((Primary OR Community OR Health) adj2 Care).ti,ab,kw,kf OR ((Health OR delivery) adj3 Care).ti,ab,kw,kf OR Healthcare.ti,ab,kw,kf OR "Health Service?".ti,ab,kw,kf OR (health* adj2 (equit* OR disparit*)).ti,ab,kw,kf	980094
9	7 OR 8	1093443
10	3 AND 6 AND 9	825
11	limit 10 to yr="2017-2022"	598

Web of Science

Date of the search: 15-11-2022
Database limit: The results of the search strategy were limited to the following 2 index: Science Citation Index Expanded (SCI-EXPANDED) & Emerging Sources Citation Index (ESCI); The results of the research strategy were limited to the last 5 years (2017-2022)

#	Search strategy	Results
1	TS=("Expert System$") OR TS=((artificial OR computational) NEAR/2 intelligence) OR TS=((Machine$ OR Supervised OR Unsupervised OR Reinforcement OR Deep) NEAR/2 Learning) OR TS=("Neural Networks") OR TS=(AI NEAR/1 (technolog* OR system$ OR algorith* OR application$)) OR TS=(predicti* NEAR/1 (model$ OR algorithm?)) OR TS="natural language processing"	730,635
2	TS=(Bias) OR TS=(biases)	521,814
3	TS=("Decision Support") OR TS=((Primary OR Community OR Health) NEAR/2 Care) OR TS=((Health OR delivery) NEAR/3 Care) OR TS=(Healthcare) OR TS=("Health Service$") OR TS=(health* NEAR/2 (equit* OR disparit*))	920,602
4	#1 AND #2 AND #3	817
5	#4 AND PY=(2017-2022)	695

CINAHL (EBSCO)

Date of the search: 15-11-2022

Database limit: The results of the research strategy were limited to the last 5 years (2017-2022)

#	Search strategy	Results
1	MH "Artificial Intelligence" OR MH "Natural Language Processing" OR MH "Expert Systems" OR MH "Neural Networks (Computer)" OR MH "Machine Learning" OR MH "Deep Learning"OR MH Algorithms OR MH "Prediction Models"	58,238
2	TI "Expert System#" OR AB "Expert System#" OR TI ((artificial OR computational) N2 intelligence) OR AB ((artificial OR computational) N2 intelligence) OR TI ((Machine# OR Supervised OR Unsupervised OR Reinforcement OR Deep) N2 Learning) OR AB ((Machine# OR Supervised OR Unsupervised OR Reinforcement OR Deep) N2 Learning) OR TI "Neural Networks" OR AB "Neural Networks" OR TI (AI N1 (technolog* OR system# OR algorith* OR application#)) OR AB (AI N1 (technolog* OR system# OR algorith* OR application#)) OR TI (predicti* N1 (model# OR algorithm#)) OR AB (predicti* N1 (model# OR algorithm#)) OR TI "natural language processing" OR AB "natural language processing"	40,870
3	S1 OR S2	85,816
4	MH "Implicit Bias" OR MH "Bias (Research)"	8,540
5	TI Bias OR AB Bias OR TI biases OR AB biases	64,322
6	S4 OR S5	69,382
7	MH "Community Health Services" OR MH "Health Care Delivery" OR MH "Primary Health Care" OR MH "Healthcare Disparities" OR MH "Decision Support Systems, Clinical" OR MH "Decision Support Techniques"	182,192
8	TI "Decision Support" OR AB "Decision Support" OR TI ((Primary OR Community OR Health) N2 Care) OR AB ((Primary OR Community OR Health) N2 Care) OR TI ((Health OR delivery) N3 Care) OR AB ((Health OR delivery) N3 Care) OR TI Healthcare OR AB Healthcare OR TI "Health Service#" OR AB "Health Service#" OR TI (health* N2 (equit* OR disparit*)) OR AB (health* N2 (equit* OR disparit*))	597,750
9	S7 OR S8	681,426
10	S3 AND S6 AND S9	298
11	S10 AND PY 2017-2022	225

PsycInfo (OVID)

Date of the search: 15-11-2022

Database limit: The results of the research strategy were limited to the last 5 years (2017-2022)

#	Search strategy	Results
1	Artificial Intelligence/ OR Natural Language Processing/ OR machine learning/ OR machine learning algorithms/ OR expert systems/ OR decision support systems/ OR neural networks/ OR artificial neural networks/ OR Algorithms/	59970
2	Expert System?.ti,ab,id OR ((artificial OR computational) adj2 intelligence).ti,ab,id OR ((Machine? OR Supervised OR Unsupervised OR Reinforcement OR Deep) adj2 Learning).ti,ab,id OR "Neural Networks".ti,ab,id OR (AI adj (technolog* OR system? OR algorith* OR application?)).ti,ab,id OR (predicti* adj (model? OR algorithm?)).ti,ab,id OR "natural language processing".ti,ab,id	44654
3	1 OR 2	79646
4	Bias.hw	16816
5	Bias.ti,ab,id OR biases.ti,ab,id	97239
6	4 OR 5	99469
7	Community Health/ OR health care services/ OR primary health care/ OR Health Care Delivery/ OR Health Disparities/ OR social equity/ OR equity/ OR Decision Support Systems/	103345
8	"Decision Support".ti,ab,id OR ((Primary OR Community OR Health) adj2 Care).ti,ab,id OR ((Health OR delivery) adj3 Care).ti,ab,id OR Healthcare.ti,ab,id OR "Health Service?".ti,ab,id OR (health* adj2 (equit* OR disparit*)).ti,ab,id	258598

9	7 OR 8	287371
10	3 AND 6 AND 9	157
11	limit 10 to yr="2017-2022"	85
