# Supplementary material for: Bias Mitigation in Primary Health Care Artificial Intelligence Models: Scoping Review
Source: J Med Internet Res. 2025 Jan 7;27:e60269. doi: 10.2196/60269 (PMC11751650; doi:10.2196/60269)
Supplement: Multimedia Appendix 2 [file jmir_v27i1e60269_app2.pdf]

Characteristics of included studies

Lead author and year/ Country/ Discipline/ Study design	Objectives	AI model features/ Data source/Model input	Model output/ Computed result of the model	Diverse groups (protected attributes) concerned	Mitigation strategies deployed	Results on protected attributes bias mitigation and on models' performance
Alday et al, 2022 **[19]**; Reyna et al, 2021**[17]** (added by handsearching) USA Informatics. Case study (a reduced-bias machine learning design model proposal)	1) To compare performance across demographics of electrocardiogram (ECG) automatic classification algorithms **[19].** 2) To propose a machine-learning method aimed at mitigating bias and enhance health equity **[19]**.	Data from various databases/recordings comprised a range of reduced-lead ECGs from patients with cardiovascular diseases **[17]**. The assessment method focused on algorithms designed for two-lead ECGs **[19].**	Open-source algorithms capable of automatically detecting cardiac abnormalities in ECG recordings using only the provided data and routine demographic information **[17].**	The data from the training, validation, and test datasets were organized based on sex (male vs. female), age (grouped by decade), and race (Asian, Black, White, and Other) when it was possible **[19].**	A constrained optimization scheme (a mathematical framework) was proposed to incorporate measures of impartiality and health equity into the objectives of machine learning design **[19].**	The AI model was retrained with an additional constraint aimed at minimizing performance disparities across sex, race, and age simultaneously. This adjustment led to a small decrease in overall performance but significantly reduced bias **[19].**
Bhanot et al, 2021 **[26]** USA<br><br>Informatics. Application of novel fairness metrics.	1) To ensure alignment between the distributions of real and synthetic data. 2) To generate synthetically data without significant deviations that could lead to	Data from three published synthetic research datasets: the American Time Use Survey (ATUS) dataset was the only primary care relevant among the three.	To generate synthetic sleep data for various age groups and *genders (*sex was identified as gender by the authors*) on the American Time Use Survey (ATUS) dataset.	Different protected attributes such as age, *gender (*sex was identified as gender by the authors*), and race.	Two fairness metrics were devised for synthetic data evaluation, examining all subgroups delineated by protected attributes to assess bias in the American Time Use Survey (ATUS) dataset.	Analysis using covariate-level disparity metrics showed potential discrepancies in the representativeness of synthetic data across both univariate and multivariate subgroup levels.

	discrimination against specific subgroups.					
Fletcher et al, 2021<br>**[27]**<br>USA<br><br>Medical Ethics and Informatics. (Case study in Pune, India).	To applicate three principles (appropriateness, bias, and fairness) using a case of machine learning pulmonary disease diagnosis and screening.	Data from a simple set of diagnostic tools (e.g., a complete battery of pulmonary function tests (PFT), which included spirometry, body plethysmography, etc).	To help predict the individual risk of several pulmonary diseases, and for the purpose of general practitioner doctors' decision support.	Age, Sex, and SES status measure or proxy: The medical care in rural India suffers from a low doctor-patient ration of 1:1700 which results in relatively high levels of underdiagnosis and misdiagnosis.	The systematic bias was examined by testing the accuracy of the model using equal size homogenous training sets.	The Allergic Rhinitis (AR) disease model showed increased stability with greater diversity in SES representation meaning that inclusivity in training data improved performance.
Foryciarz et al, 2022<br>**[28]**<br>USA<br><br>Informatics.	1) To measure the impact of two fairness methods, 2) To demonstrate principles applicable for conducting contextually pertinent fairness assessments of models used in clinical settings,	Data from an updated dataset from pooled cohorts focusing on 10-year Atherosclerotic Cardiovascular Disease (ASCVD) risk predictions was used.	The 10-year Atherosclerotic Cardiovascular Disease (ASCVD) risk predictions aim to inform a clinician-patient shared decision-making on initiating statin therapy.	Current ASCVD models do not inherently account for variations in race, ethnicity, and *gender-specific groups.<br>*(sex was identified as gender by the authors).	Two algorithmic fairness strategies—group recalibration and equalized odds—was examined as means to refine risk estimations, ensuring they align with the underlying assumptions of the decision rules within the guidelines.	Compared with an unconstrained model, group-recalibration enhances calibration at specific thresholds for each group but exacerbates differences in false positive and false negative rates between groups. An equalized odds constraint, aimed at balancing error rates across groups, achieves this but misalign the overall model.
Ghai and Mueller, 2022<br>**[29]**<br>USA<br><br>Informatics. Case study.	To develop and propose a visual interactive tool (*D-BIAS*) that embodies human-in-the-loop AI approach for	The Adult Income dataset was used as a tool for bias identification and mitigation. A random sample of 3000	The prediction task of this AI model is to classify if a person's income will be greater or lesser than	Age, work class, education, marital status, race, *gender (*sex was identified as gender by the authors*), and income.	The tool was evaluated by experimenting three datasets and a formal user study.	The tool significantly reduces bias compared to the baseline method across various fairness metrics with minimal data distortion and slight utility loss. The human-

	auditing and mitigating social biases from tabular datasets.	points was used for faster computation.	$50k/year based on their personal attributes.			in-the-loop approach notably surpasses other methods in trust, interpretability, and accountability.
Hane and Wasserman, 2022 **[30]** USA<br><br>Informatics.	To implement practical tools aimed at facilitating the fairer utilization of risk scores in outreach programs.	From a data set of 1,511,260 members in a commercial or Medicare Advantage plan from the "*Optum Labs Data Warehouse (OLDW)*".	To predict next year's inpatient stays. Risk scores aims to select which patients will receive aid and support.	Age, *gender (*sex was identified as gender by the authors*), and race/ethnicity.	Pragmatic tools were proposed to a fairer use of risk scores. The method output charts allow users to select the optimal risk thresholds to trigger outreach.	These tools aid stakeholders in seeing the suitable risk score thresholds for different patient groups to ensure Equality of Opportunity (EOp). They are applicable regardless of the machine learning or statistical model used in score generation.
Juhn et al, 2022 **[31]** USA<br><br>Medical informatics. Case study.	To evaluate how disparities in data quality within electronic health records (EHRs) impact the varying performance of AI models across different socioeconomic status (SES) levels.	Data from a prior study included the training of two machine learning models. Variables, such as sociodemographic factors, risk factors, and asthma outcomes, were extracted from electronic health records (EHRs) over a preceding three-year period.	To estimate 1-year asthma exacerbation (AE) risk among children with asthma.	Authors focused to quantify bias in model performance by socioeconomic status (SES), and considered other readily available demographic characteristics (e.g., age, sex, and race/ethnicity), and pediatric chronic conditions.	The balanced error rate (BER) across various SES levels was compared and assessed using the HOUsing-based SocioEconomic Status measure (HOUSES) index, along with the incompleteness of EHR information on asthma care in relation to SES as a potential source of bias.	Children with asthma from lower SES backgrounds exhibited higher BER compared to those from higher SES backgrounds, with a notable ratio difference Also, they had a greater proportion of missing information relevant to asthma care, such as missing asthma severity and undiagnosed asthma despite meeting asthma criteria.
Khurshid et al, 2022 **[32]** USA	To develop a multi-institutional EHR cohort named "Community Care	Data from 520,868 individuals aged 18–90 who received regular primary care, with at least two	To predict more accurately cardiovascular disease risk with C3PO than for	Potential racial discrimination examining four categories: (White Women, Black	Development and implementation of a deep natural language processing (NLP) model by	NLP helped find missing vital signs in EHR by 31%. C3PO risk models worked better compared to the Convenience Samples. By

Medical Informatics.	Cohort Project (C3PO)" with a focus on cardiovascular disease with two main objectives: (1) To mitigate ascertainment bias and (2) reduce data missingness.	visits within 1–3 consecutive years.	Convenience Samples Models such as Pooled cohort equations (PCE) or Cohorts for Heart and Aging Research in Genomic Epidemiology for Atrial Fibrillation (CHARGE-AF).	Women, White Men, Black Men) in C3PO versus Convenience Samples.	extracting four vital sign features from unstructured notes, and evaluation of effectiveness by comparing sample sizes before and after missing data recovery.	looking at patients who regularly visit their primary care doctor and using NLP to find missing information, prediction can be apply it to more people and therefore in fairness.
Martinez-Martin et al, 2021 **[33]** USA<br><br>Medical Ethics. Delphi study.	To establish consensus statements on fundamental ethical principles guiding the use of digital phenotyping in mental health applications within the United States.	Digital phenotyping is studying behavior using data from digital devices. Data includes information collected in real-life settings. By monitoring things such as pulse rate or finger taps, or voice characteristics,	To assess behavior, physical health, and cognitive performance continuously. This helps in understanding someone's mental state or predicting their future actions.	Data streams may not adequately include people of different racial, socioeconomic, or disability status.	Delphi study was used to address ethical issues raised by mental health applications of digital phenotyping, such as privacy and data protection, consent, transparency, potential for bias in outcomes, and accountability.	This study revealed a consensus on ethical concerns concerning the development of mental health apps using digital phenotyping, including privacy, transparency, consent, accountability, and fairness.
Nong et al, 2022 **[34]** USA<br><br>Management Care Ethics. Qualitative analysis of user-centered design (n=46) and expert interviews (n=10).	To (1) identify user requirements for informed decision-making and utilization of predictive models and (2) anticipate and reflect equity concerns in the information provided about models.	This is an exploratory study based in a single academic institution (interviews were conducted at a large medical institution with predictive analytics infrastructure).	Semi structured interviews were conducted to understand participants' needs, concerns, and preferences related to a hypothetical hepatitis C model prototype that predicted serious illness to allocate	All minority groups susceptible to disparities in treatment access, such as racial and ethnic minorities.	A user-centered design study at an academic medical center with clinicians and stakeholders to identify elements required for decision-making related to predictive models. Equity focused interviews with experts were also conducted.	Four elements were identified: details of (1) the model's developers and users, (2) the methodology employed, (3) the model's peer review processes and updates, and (4) the model's validation with different populations. Equity-related concerns raised in interviews with experts focused on the purpose or application of

			treatment resources.			the model and its link to systemic inequalities.
Obermayer et al, 2019 **[35]** USA<br><br>Data Science	To investigate the potential racial bias of a widely used algorithm that affects patients, particularly black patients, who when at risk scores similar to white patients, appear to have more severe health conditions.	Data from all primary care patients enrolled in risk-based contracts from 2013 to 2015 in a large academic hospital. The main sample consisted of 6079 patients who self-identified as Black and 43,539 patients who self-identified as White without another race or ethnicity.	The stated aim of the algorithm studied was to predict individuals with higher health needs to offer interventions and resources to meet those needs.	Race: Black patients versus White patients.<br>The algorithm was taking a large set of raw insurance claims data over a year. However, the algorithm specifically excluded race.	The existing model infrastructure (excluding race, as before), was used but the label was changed. Rather than future cost, an index variable that combined health prediction with cost prediction was created.	The bias arises because the algorithm predicts health care costs rather than illness, but unequal access to care means that less money is spending caring for Black patients than for White patients. Remedying this disparity would increase the percentage of Black patients receiving additional help from 17.7 to 46.5%.
Panigutti et al, 2021 **[36]** Italy<br><br>Medical Informatics. Use case.	To present a tool, named "FairLens", that can audit black-box model acting as a clinical decision support system (DSS).	Data from the MIMIC-IV (Medical Information Mart for Intensive Care) database. Although not primary care dataset focused, MIMIC may still be pertinent for primary care clinicians.	A fictional clinical decision support system (DSS). Most health systems used these programs considering effective at improving outcomes while reducing costs.	This tool presents patient data based on demographic attributes such as age, ethnicity, *sex (*identified as gender), and health insurance (SES proxy).	The reliability of this tool in discovering biases was tested through a fictional commercial biased black-box model named "*Doctor AI*".	"FairLens" could reveal biases injected into the fictitious DSS when other standard multi-label performance measures failed to detect them. Experts could explore a particular misclassification by identifying elements in the clinical history of patients in the groups concerned.
Park et al, 2022 **[20]**<br>Singh and Long, 2018 **[18]** (added by handsearching). USA	To 1) analyze the susceptibility of commonly used machine learning approaches for sex bias in mobile mental health assessment; 2)	Using the data set (n=55) obtained in a previous study **[18]**, preprocessing and model training were carried out **[20]**. From a sample of 55 participants, 21	To detect and predict mental health problems (automated ML algorithms used the smartphone's characteristics, automatically	Accuracy levels and differences in accuracy across **genders* (*sex was identified as gender by the authors*), were computed using five different	Random forest model, which yielded the highest accuracy, was selected for a more detailed audit, and computed multiple metrics that are commonly used	The highest accuracy observed for mental health assessment was 78.57%. However, auditing based on *gender [sex as gender] revealed that performance was

Informatics.	explore the use of an algorithmic disparate impact remover (DIR) approach to reduce bias levels while maintaining high accuracy **[20]**.	(38%) declared themselves to be women or female (minority class), and 34 (62%) described themselves as man or male **[18]**.	classifying a person's level of mental health and general well-being with an accuracy of around 80% **[18]**.	machine learning models **[20]**.	for fairness in the machine learning literature. Then, the disparate impact remover (DIR) approach was applied to reduce bias in the algorithm **[20]**.	statistically higher for males than females. This disparity was considerably reduced after applying the DIR approach by adjusting the data used for modelling **[20]**.
Park et al, 2021 **[37]** USA<br><br>Medical Informatics. Cohort study.	To assess methods for mitigating bias in machine learning models within a practical clinical context.	Based on data from a cohort built using the "IBM MarketScan Medicaid database" (2014-2018), containing de-identified claims records from around seven million Medicaid enrollees in several US states.	Prediction of 2 binary outcomes: postpartum depression (PPD) and postpartum mental health service utilization.	Binary race (black and white individuals): cohort study including 314,903 white pregnant women and 217,899 black pregnant women with Medicaid coverage.	A 3-fold approach: 1) Reweighing, a preprocessing method; 2) Prejudice Remover for logistic regression, an in-processing method; and 3) Models training without the race variable for comparison, an approach known as fairness through unawareness.	A reweighing method was associated with a greater reduction in algorithmic bias for postpartum depression and mental health service utilization prediction between white and black pregnant women than simply excluding race from the prediction models.
Seker et al, 2022 **[38]** USA<br><br>Medical Informatics.	To examine bias within electronic health record (EHR) data by: (1) measuring the level of discrimination, (2) addressing bias through re-balancing class labels, and (3) evaluating and comparing the modeling outcomes before	An integrated dataset was created by adding zip code-level information to 19,367 electronic health records (EHRs) of patients diagnosed with chronic diseases (including asthma, diabetes, various heart conditions, stroke, etc.) sourced from the University of Arkansas for Medical Sciences	Patients are prioritized based on their risk level (multimorbidity) to ensure they receive care tailored to their specific needs. A simplified risk prediction model was developed to examine the effect of preprocessing data to mitigate	Urban and rural residency status. (Place of residence) was identified as a potential bias against rural patients in algorithms using diagnostic data for risk assessment.	The level of discrimination favoring metropolitan patients in multimorbidity classification were measured in a model using the biased data to determine optimal classification performance. Then, a new training dataset and model, without bias, were developed	The classification performance of the new model on unchanged data showed no significant deviation from the performance of the initial optimal model trained on biased data, indicating that bias can be effectively mitigated through preprocessing.

	and after processing.	Clinical Data Warehouse.	bias on predictions.		and assessed against unchanged validation data.	
Straw and Wu, 2022 **[39]** UK<br><br>Medical Informatics.	Following a literature review of the Indian Liver Patient Dataset (ILPD) publications: To replicate the machine learning (ML) models from these previous studies and subsequently examine them for bias.	The ILPD was originally collected from India and consists of 583 patient records, of which 416 have liver disease. The ILPD was imported from the *"UCI machine learning Repository, Irvine, Ca."*.	To diagnose liver diseases: Predictive machine learning models may benefit patient care if liver diseases can be diagnosed at an earlier stage.	Unisex biochemical thresholds used in research (based only on males' features) may disadvantage female patients in practice.	Four experiments trained on sex-unbalanced/balanced data, with and without feature selection. Random forests (RFs), support vector machines (SVMs), Gaussian Naïve Bayes and logistic regression (LR) classifiers were built, running experiments 100 times, and reporting average results with Standard deviation.	Published models were reproduced achieving accuracies of >70% and demonstrated a previously unobserved performance disparity. Across all classifiers females suffer from a higher false negative rate (FNR). RF and LR classifiers are reported as the most effective models. However, in their experiments they demonstrated the greatest false negative rate (FNR) disparity.
Yan et al, 2022 **[40]** USA<br><br>Medical Informatics.	To illustrate the impact of observability on differential bias for a clinical prediction model (CPM).	Data of all discharges were collected in years 2018-2019 from an hospital EHRs. Development of a simple 30-day readmission CPM using age, length of stay, admission source, and five comorbidities.	Development of a simple 30-day readmission CPM using age, length of stay, admission source, and five comorbidities.	Place of residence: Each patient was categorized being local (within the hospital's county) or non-local.	The CPM performance was compared with and without this variable (of being local or not). Differences in observing an outcome that may induce differential bias were investigated.	There is not a meaningful difference between the local and non-local groups. Living locally only impacting the observability of the outcome (i.e., 30-day readmission) and cannot determine if there is a differential bias or not.
